# An International Comparison of Women's Health Issues in the Philippines, Thailand, Malaysia, Canada, Hong Kong, and Singapore: The CIDA-SEAGEP Study

**DOI:** 10.1100/tsw.2004.201

**Published:** 2004-11-19

**Authors:** Bernard Choi

**Affiliations:** Department of Public Health Sciences, University of Toronto and Department of Epidemiology and Community Medicine, University of Ottawa, 432 Pleasant Park Road, Ottawa, Ontario, Canada K1H 5N1,

**Keywords:** women’s health, developing countries, developed countries, surveillance, research, programs, policies

## Abstract

This was an international study of women's health issues, based on an Official Study Tour in Southeast Asia (the Philippines, Thailand, Malaysia, Hong Kong, and Singapore) and Canada. The objectives of the study were to identify and compare current gaps in surveillance, research, and programs and policies, and to predict trends of women''s health issues in developing countries based on the experience of developed countries. Key informant interviews (senior government officials, university researchers, and local experts), self-administered questionnaires, courtesy calls, and literature searches were used to collect data. The participating countries identified women's health as an important issue, especially for reproductive health (developing countries) and senior's health (developed countries). Cancer, lack of physical activity, high blood pressure, diabetes, poverty, social support, caring role for family, and informing, educating, and empowering people about women's health issues were the main concerns. Based on this study, 17 recommendations were made on surveillance, research, and programs and policies. A number of forthcoming changes in women's health patterns in developing countries were also predicted.

## BACKGROUND

The health of people in the world is improving. Their life expectancy is lengthening. However, our health system has not always understood the factors that influence the health status of women, nor has it addressed women's health issues adequately[1]. Women's health issues are complex[1,2,3,4,5,6,

*The views expressed in this paper are solely those of the author, and do not necessarily represent those of the University of Toronto, University of Ottawa, or any agencies or organizations.
©2004 with author.

7,8,9,10,11], in particular due to their biological (sex) and social (gender) dimensions[1,2,3]. A wake-up call is needed to advance women's health[12] and to identify priorities for women's health[13].

This paper describes the findings of a study funded by the Canadian International Development Agency's Southeast Asia Gender Equity Program (CIDA-SEAGEP). The main purpose of the study was to undertake an international comparison of the situations on women's health issues to find out if there are gaps in women's health surveillance, research, and programs and policies. The gaps were studied through the use of health, risk, and intervention indicators, as suggested by Choi[14]. A second purpose was to identify emerging women's health issues and see if there is a trend from developing countries to developed countries. If a trend is observed, the study might help developing countries in Southeast Asia to predict the type of women's health issues that may soon occur due to globalization of the economy.

## METHODS

The international comparison study had four components: key informant interviews, self-administered questionnaires, courtesy calls, and literature research. Key informant interviews were conducted for the Philippines, Thailand, Malaysia, Canada, Hong Kong (China), and Singapore during an Official Study Tour of Southeast Asia in August and September, 2000 and Canada in October, 2000. Self-administered questionnaires were completed and mailed back by the office of the key informants. Courtesy calls were made to selected high-ranking officials. Literature research was conducted through MEDLINE and the Internet.

The participating countries were selected because of their developing (the Philippines, Thailand, and Malaysia) and developed (Canada, Hong Kong, and Singapore) status in economy, and because CIDA-SEAGEP has a particular interest in Southeast Asia. The developing and developed status was defined according to the criterion of the World Bank[15,16,17,18].

All key informants were interviewed in their own country, except for the Philippines for which the key informants were interviewed during a women's health conference in Hong Kong. Interviews were conducted in English, but where necessary, local professional interpreters were used. The Official Study Tour covered the following places: Bangkok, Nonthaburi, Kuala Lumpur, Shah Alam, Ottawa, Toronto, Hong Kong, and Singapore.

The Official Study Tour was arranged through the CIDA-SEAGEP headquarters in Singapore, CIDA-SEAGEP posts in the other Southeast Asian countries, and the Canadian embassy in Thailand. Key informants were identified for four categories: (1) senior officials in the ministry of health, (2) senior officials in the ministry of labor, (3) senior researchers (public health or occupational health) in the university, (4) local experts in public health, occupational health, and women's health. Key informants were identified through suggestions from the ministries of health and labor and local women's health experts of the participating countries, and the author's own professional contacts.

The key informant interview was conducted using a structured questionnaire that consisted of open-ended (narrative) and close-ended (checklist) questions. The interview was short (approximately 20 min) because of the limited time available from the respondents. The questionnaire first asked the key informants to give, narratively, three reasons for the importance, and three important examples of specific initiatives (programs), diseases (health conditions), risk factors (biological and physical), social determinants (social and economic), and challenges (difficulties) of women's health issues in their countries. The same questions were then asked a second time, with a checklist, to ensure completeness and consistency in the responses.

After the interview, a detailed, self-administered questionnaire was given to the key informant for their staff to locate national statistics from local sources and publications. The self-administered questionnaire was to be mailed back within 1 month.

Local courtesy calls were made to high-ranking officials in various government departments, international agencies, research communities, and advocacy groups. Extensive notes were taken, but no questionnaire was used.

The basic data analysis was cross-tabulation of information by country, arranged in the order of increasing per capita income of the country in 1999. This allowed identification of trends, if any, from developing countries to developed countries. Analysis of opinion from the key informant interviews and courtesy calls was performed using a summarizing technique developed by Choi[19,20,21,22,23]. The technique uses a mark, e.g., a circle[21], a cross[22], combination of circle and cross[19], a black dot[23], or a directional arrow[20] to indicate the presence or absence, and the direction of effect, of an attribute. In this study, a black dot was used to summarize the responses for each country. An attribute was considered to be important for the country (black dot) if the key informants were consistent in their narrative and checklist responses, and if more than 50% of the key informants in that country agreed that it was important. Detailed notes taken during courtesy calls were also used to assist on the decision on the black dot.

## RESULTS

### Development Status of the Participating Countries

World Bank development indicators for the participating countries are shown in Table 1. Using the World Bank criterion that developing countries have 1999 gross national product (GNP) per capita of US$9,265 or less and developed countries have US$9,266 or more, the Philippines, Thailand, and Malaysia were considered developing countries, whereas Canada, Hong Kong, and Singapore were developed countries. For operational and analytical purposes, starting in 2000, the World Bank's main criterion for classifying economies is gross national income (GNI) per capita. Based on GNI per capita, the Philippines and Thailand were lower middle income, Malaysia was upper middle income, and Canada, Hong Kong, and Singapore were high income. Other analytical groups, based on levels of external debt and geographic regions, are also available from the World Bank (Table 1).

### Key Informant Interviews and Courtesy Calls

All individuals contacted for the key informant interview and courtesy call agreed and participated in the study (response rate 100%). The number of key informant interviews was as follows: the Philippines 3, Thailand 5, Malaysia 3, Canada 9, Hong Kong 3, and Singapore 4, a total of 27 interviews. Their background was as follows: ministry of health (director, senior advisor) 9, ministry of labor (director, senior advisor) 5, university (dean, professor) 8, research institute and nongovernment organization (director, senior scientist) 5. The number of courtesy calls was as follows: the Philippines 0, Thailand 8, Malaysia 0, Canada 3, Hong Kong 3, and Singapore 2, a total of 16 courtesy calls.

Table 2 shows the important diseases and health conditions considered to be affecting women's health in the various countries. All six participating countries considered cancer (in particular breast and cervical) to be important for women's health. Five countries considered the following to be important for women: diabetes, osteoporosis, reproductive disorder, and menopause-related condition. Four countries considered the following to be important: mental health, human immunodeficiency virus (HIV), acquired immunodeficiency syndrome (AIDS), sexually transmitted disease (STD), suicide attempts, cardiovascular disease (CVD), and musculoskeletal disorder. No country considered multiple chemical sensitivities (MCS) or fibromyalgia (chronic pain) to be important.

Table 2 also shows the top five major causes of death among women according to national health statistics in 1999 (based on data from the returned questionnaires). Most of the top five major causes of death among women were mentioned by the key informants as important diseases and health conditions for women. However, in several countries, it did not occur to more than 50% of key informants at the time of interview that lung disease and injury/accident (top five major causes) were important diseases and health conditions affecting women's health (Table 2).

**TABLE 1**
**World Bank Development Indicators, by Country/Region***

| | Philippines | Thailand | Malaysia | Canada | Hong Kong | Singapore |
|---|---|---|---|---|---|---|
| 1999 GNP per capita (US$)[1] | 1,020 | 1,960 | 3,400 | 19,320 | 23,520 | 29,610 |
| 2000 GNI per capita (US$)[2] | 1,040 | 2,010 | 3,370 | 21,050 | 25,950 | 24,740 |
| Development status[1] | Developing | Developing | Developing | Developed | Developed | Developed |
| Income group[3] | Lower middle income | Lower middle income | Upper middle income | High income: OECD | High income: non-OECD | High income: non-OECD |
| Indebtedness[4] | Moderately indebted | Moderately indebted | Moderately indebted | Debt not classified | Debt not classified | Debt not classified |
| World Bank geographic region[5] | East Asia and Pacific | East Asia and Pacific | East Asia and Pacific | — | — | — |

* "—" indicates not applicable.

[1]GNP, gross national product. Developing countries have 1999 GNP per capita of US$ 9,265 or less, while developed countries have US$ 9,266 or more [16].
[2]GNI, gross national income (the new terminology for gross national product used by the World Bank, starting in 2000) [17].
[3]Income group is based on GNI per capita in 2000: low income US$755 or less; lower middle income US$756–2,995; upper middle income US$2,996–9,265; high income US$9,266 or more. OECD, organization for economic cooperation and development, which has 30 member countries sharing a commitment to democratic government and the market economy[18].
[4]Standard World Bank definitions of severe and moderate indebtedness are used to classify economies. Debts of high-income economies are not classified[18].
[5]World Bank classification for geographic regions are for low-income and middle-income economies only[18].

All six countries considered three risk factors (biological and physical) to be important for women's health: lack of physical activity, high blood pressure, and diabetes (Table 3). Five countries considered obesity, and four countries considered smoking, poor nutrition, environmental conditions, and occupational exposures to be important. No country considered school conditions to be important.

Regarding social determinants (social and economic), five countries considered poverty to be an important issue (Table 4). Other important issues included caring role for family and friends, poor housing, single motherhood, violence against women, and vulnerability to unemployment. Important challenges (difficulties) in women's health included informing, monitoring, investigation, research, and funding (Table 5).

## The Self-Administered Questionnaire and Literature Research

Self-administered questionnaires and literature research provided relevant national statistics. The participating countries differ geographically, economically, demographically, and health-wise (Table 6). For example, Singapore and Hong Kong have an area of 620 and 1070 $km^2$ and a very high population density of 6,479 and 6,770/$km^2$, respectively. On the other hand, Canada has a vast area of close to 10 million $km^2$ and a population density of only 3/$km^2$.

## TABLE 2

### Opinion on Important Diseases and Health Conditions Affecting Women's Health, According to Key Informants During Interview, by Country/Region*

| Disease / health condition | Philippines | Thailand | Malaysia | Canada | Hong Kong | Singapore |
|---|---|---|---|---|---|---|
| Cancer (breast, cervical) | ●(IV) | ●(II) | ● | ●(II) | ●(I) | ●(I) |
| Diabetes | | ● | ● | ● | ● | ● |
| Osteoporosis | | ● | ● | ● | ● | ● |
| Reproductive disorder/ abnormal pregnancy | ● | ● | ● | ● | ● | |
| Menopause related condition | ● | ● | ● | ● | ● | |
| Mental health | | ● | ● | ● | ● | |
| Human immuno-deficiency virus (HIV)/ Acquired immunodeficiency syndrome (AIDS) | | ● | ● | ● | ● | |
| Sexually transmitted disease (STD) | | ● | ● | ● | ● | |
| Suicide attempts | | ● | ● | ● | ● | |
| Cardio-vascular disease | ●(I) | ●(I) | | ●(I) | ●(II) | (II) |
| Musculo-skeletal disorder | ● | ● | ● | ● | | |
| Lung disease | (II) | (III) | ● | ●(III) | ●(III) | (III) |
| Communicable disease | ●(III) | ●(V) | | | ● | |
| Injury/ accident | (V) | ●(IV) | ● | ●(IV) | (V) | (V) |
| Occupational disease | | ● | ● | ● | | |
| Car accident | ● | ● | ● | | | |
| Dental health | ● | ● | ● | | | |
| Eating related disorder | | | ● | ● | | |
| Occupational injury/ accident | | ● | ● | | | |
| Chronic fatigue syndrome (CFS) | ● | | | | | |
| Multiple chemical sensitivities (MCS) | | | | | | |
| Fibromyalgia (FM)/ chronic pain | | | | | | |

*A dot indicates that more than 50% of the key informants in that country agreed that it was an important disease or condition; the Roman numeral in brackets indicates (according to national health statistics in 1999) the ranking of the disease or condition as a major cause of death among women.

**TABLE 3**
**Opinion on Important Risk Factors (Biological and Physical) Affecting Women's Health, According to Key Informants During Interview, by Country/Region**

| | Philippines | Thailand | Malaysia | Canada | Hong Kong | Singapore |
|---|---|---|---|---|---|---|
| Lack of physical activity | ● | ● | ● | ● | ● | ● |
| High blood pressure | ● | ● | ● | ● | ● | ● |
| Diabetes | ● | ● | ● | ● | ● | ● |
| Obesity | | ● | ● | ● | ● | ● |
| Smoking | ● | | | ● | ● | ● |
| Poor nutrition | | ● | ● | ● | ● | |
| Environmental conditions | ● | ● | ● | | ● | |
| Occupational exposures | ● | ● | ● | | ● | |
| Air and water quality | ● | ● | ● | | | |
| Access to pap test | | | | ● | ● | |
| Drug addiction/abuse | | ● | | | ● | |
| Contraception | | ● | | ● | | |
| Abortion | | ● | ● | | | |
| Alcohol addiction/abuse | | | | | ● | |
| Access to mammography | | ● | | | | |
| School conditions | | | | | | |

When compared according to the World Bank development indicators, many of the economy, demography, and health indicators showed a trend. In the order of the Philippines, Thailand, Malaysia, Canada, Hong Kong, and Singapore (according to GNP per capita), there was an increasing trend in electricity power consumption, telephone mainlines, personal computers, Internet hosts, and urban population and a decreasing trend in poverty (Table 6). There was no apparent trend in illiteracy rate or school enrollment.

Comparing females to males, the female illiteracy rate was in general higher, by as much as two- to threefold, in both the developing and developed countries (Table 6). There were slightly decreased female primary school enrollment rates compared to the males in the Philippines, Thailand, and Singapore, but increased female secondary school enrollment rates in the Philippines, Thailand, and Malaysia. The female population represented about 50% of the total population (from 49.4% in Malaysia to 50.4% in Canada).

Concerning health, there was an increase in life expectancy with the development status of the countries, for example, from 69 years in the Philippines to 78 years in Singapore (a 9-year difference) and an increase in contraceptive prevalence (47% in the Philippines to 72% in Hong Kong) (Table 6). There was a decrease in fertility rate (3.5 births per women in the Philippines to 1.5 births per women in Singapore), infant mortality rate (43.5 deaths per 1,000 live births in the Philippines to 4.1 deaths per 1000 live births in Singapore), and crude birth rate (from 27 births per 1,000 people in the Philippines to 13 births per 1,000 people in Singapore).

Comparing females to males, the female life expectancy exceeded the male life expectancy by about 3–5 years (Table 6). The excess female life expectancy was not related to the economy, as the female to male life expectancy ratio was the highest in Canada (1.08) and the lowest in Singapore (1.05), both

**TABLE 4**
**Opinion on Important Social Determinants (Social and Economic) Affecting Women's Health, According to Key Informants During Interview, by Country/Region**

| | Philippines | Thailand | Malaysia | Canada | Hong Kong | Singapore |
|---|---|---|---|---|---|---|
| Poverty/low income | ● | ● | ● | ● | ● | |
| Caring role for family and friends | ● | | ● | ● | ● | |
| Poor housing | ● | ● | ● | ● | | |
| Single mother/lone parenthood | | | ● | ● | ● | |
| Violence against women | | ● | | ● | ● | |
| Vulnerable to unemployment | ● | ● | | ● | | |
| Differential access to healthcare | | ● | | ● | | |
| Not included in health research | | | | ● | | |
| Not included in clinical studies of medication | | | | ● | | |
| Minority/aboriginal issue | | | | ● | | |
| Domestic problem | | | ● | | | |
| Poor working condition | | ● | | | | |
| Not included in clinical studies of medical devices | | | | | | |
| Greater difficulties in employment | | | | | | |
| Fewer legislations to protect women's rights | | | | | | |

considered developed countries. Females also had a health advantage compared to males in infant mortality rate and all-cause mortality.

In developed countries, major causes of death among females were chronic diseases such as heart diseases, cancer, respiratory diseases, and injury. In developing countries, infectious diseases played an increased role as a cause of death (Table 6). In Canada and Singapore, where relevant statistics were available, females had a longer hospital stay than males for heart, respiratory, digestive and musculoskeletal diseases, and injury; but taking all diseases together, females had a shorter hospital stay than males (Table 6).

Female smoking is a problem in Canada (smoking prevalence 23%), but in general not a problem in Southeast Asia (2–3%). However, data indicated an increase in female smoking prevalence in the 15–34 year age group compared with other age groups (Table 6).

## DISCUSSION

The World Bank has indicated that the use of the term "developing" or "developed" is convenient; it is not intended to imply that all economies in the group are experiencing similar development or that some economies have reached a preferred or final stage of development[15,16,17,18]. This study found that

**TABLE 5**
**Opinion on Important Challenges (Difficulties) Affecting Women's Health, According to Key Informants During Interview, By Country**

| | Philippines | Thailand | Malaysia | Canada | Hong Kong | Singapore |
|---|---|---|---|---|---|---|
| Informing, educating and empowering people about women's health issues | | ● | ● | ● | ● | ● |
| Monitoring women's health status | ● | | | ● | ● | |
| Investigation of women's health problems and hazards | ● | | | ● | ● | |
| Conducting research for new insights and innovative solutions for women's health | ● | ● | | ● | | |
| Adequate funding for projects and programs | ● | ● | | ● | | |
| Mobilizing partners to identify and resolve women's health problems | | | | ● | ● | |
| Enforcing laws and regulations that protect women's health and safety | | ● | | | | |
| Communication through the media | ● | | | | | |

classification of a country’s economy by income is a useful way to help understand women’s health issues.

Women's health is an emerging, but difficult area in public health. As one key informant put it, “there are no formal statistics, therefore one can only get anecdotal accounts and sniff around to get to the truth.” This Official Study Tour represented such an effort. A large number of anecdotes and stories were collected first hand on women's health issues.

To increase the validity of the information collected, this study made a number of methodological improvements. First, selection of respondents for interviews and courtesy calls were made according to four predetermined categories (ministry of health, ministry of labor, university, and local expert) in order to increase representativeness. Second, a structured questionnaire was used to increase uniformity and comparability of information. Third, the key informant interview was brief (about 20 min) to avoid response fatigue bias[24], with the brevity compensated by a detailed questionnaire mailed back within 1 month. Fourth, during the interview, the key informants were first asked to give narrative answers, because as senior officials or researchers what they had in mind (which do not necessarily reflect the true situation) was the driving force of government and research policies. This was then followed by a checklist to ensure completeness and consistency in the answers. Fifth, the subsequent returned questionnaire provided objective national data for comparison with the subjective opinion expressed by the key informants. Sixth, the study was supplemented by information from a number of courtesy calls. Courtesy calls were conducted instead of formal interviews because of logistics and cultural reasons. For example, some respondents were very high-ranking government officials who, according to local practices, should not be interviewed using a structured questionnaire.

There are limitations of this study, including its small sample size, a semi-qualitative nature, and therefore inability to apply proper statistical analysis such as test of significance, trend analysis, or control for confounding. As a result, this study can only identify broad issues and cannot provide in-depth analysis or definitive evidence. Despite limitations, a number of emerging women’s health issues were identified as follows:

## TABLE 6
**National Statistics Provided by Key Informants in the Returned Questionnaire and by Literature search, by Country/Region, Based on Most Recent Data Available at the Time of the Study (2000)***

| | Philippines | Thailand | Malaysia | Canada | Hong Kong | Singapore |
|---|---|---|---|---|---|---|
| *Geography* | | | | | | |
| Area (sq km) | 300,000 | 513,120 | 329,750 | 9,970,610 | 1,070 | 620 |
| *Economy* | | | | | | |
| Electricity power consumption (kwh per capita) | 451 | 1,345 | 2,554 | 15,000 | 5,244 | 6,672 |
| Telephone mainlines (per 1,000 people) | 39 | 84 | 203 | 655 | 576 | 482 |
| Personal computers (per 1,000 people) | 17 | 23 | 69 | 361 | 298 | 437 |
| Internet hosts (per 10,000 people) | 1 | 4 | 24 | 425 | 146 | 263 |
| Poverty (% of population below national poverty line) | 37 | 16 | 8 | — | — | — |
| *Demography* | | | | | | |
| Total population (millions) | 74.3 | 60.3 | 22.7 | 30.5 | 6.7 | 3.9 |
| Female population (% of total) | 49.6 | 50.1 | 49.4 | 50.4 | 50.0 | 49.7 |
| Population density (people per km$^2$) | 249 | 118 | 69 | 3 | 6,770 | 6,479 |
| Urban population (% of total population) | 59 | 20 | 57 | 77 | 100 | 100 |
| Illiteracy rate (% of population ages 15+) | 5 | 5 | 13 | — | 7 | 8 |
| Female illiteracy rate (% of females ages 15+) | 5 | 7 | 17 | — | 10 | 12 |
| Male illiteracy rate (% of males ages 15+) | 5 | 3 | 9 | — | 4 | 4 |
| School enrollment, primary (% of school-age population) | 98 | 77 | 98 | 97 | 94 | 94 |
| Female school enrollment, primary (% of school-age females) | 97 | 76 | 98 | 97 | 95 | 93 |
| Male school enrollment, primary (% of school-age males) | 99 | 78 | 98 | 97 | 93 | 95 |
| School enrollment, secondary (% of school-age population) | 50 | 55 | 93 | — | — | — |
| Female school enrollment, secondary (% of school-age population) | 52 | 57 | 97 | — | — | — |
| Male school enrollment, secondary (% of school-age population) | 48 | 53 | 89 | — | — | — |
| *Health* | | | | | | |
| Life expectancy at birth (years) | 69 | 69 | 72 | 79 | 80 | 78 |
| Female life expectancy at birth (years) | 70 | 72 | 75 | 81 | 82 | 80 |

**TABLE 6 (continued)**

| | Philippines | Thailand | Malaysia | Canada | Hong Kong | Singapore |
|---|---|---|---|---|---|---|
| Male life expectancy at birth (years) | 67 | 68 | 70 | 76 | 77 | 76 |
| Life expectancy; female to male ratio | 1.06 | 1.06 | 1.07 | 1.08 | 1.07 | 1.05 |
| Contraceptive prevalence (% of women ages 15–49) | 47 | 65 | 51 | 73 | 72 | — |
| Fertility rate (births per women) | 3.5 | 1.9 | 3.0 | 1.6 | 1.3 | 1.5 |
| Infant mortality rate (under 1 year/1,000 live births) | 43.5 | 31.5 | 8.0 | 5.6 | 3.2 | 4.1 |
| Female infant mortality rate (under 1 year/1,000 live births) | 38.0 | 18.2 | — | 5.5 | 2.9 | 4.1 |
| Male infant mortality rate (under 1 year/1,000 live births) | 49.0 | 20.8 | — | 6.7 | 3.5 | 4.1 |
| Infant mortality; female to male ratio | 0.78 | 0.88 | — | 0.82 | 0.83 | 1.00 |
| Crude birth rate (per 1,000 people) | 27 | 17 | 24 | 11 | 8 | 13 |
| Female all-cause mortality (per 100,000 people) | 447.3 | 428.6 | (520.0) | 528.2 | 419.9 | 408.9 |
| Male all-cause mortality (per 100,000 people) | 652.9 | 611.5 | (520.0) | 854.7 | 565.0 | 494.7 |
| All-cause mortality; female to male ratio | 0.69 | 0.70 | — | 0.62 | 0.74 | 0.83 |
| Female major causes of death | Heart<br>Pneumonia<br>Tuberculosis<br>Cancer<br>Injury | Heart<br>Cancer<br>Respiratory<br>Injury<br>Infectious | — | Heart<br>Cancer<br>Respiratory<br>Injury<br>Digestive | Cancer<br>Heart<br>Pneumonia<br>Stroke<br>Injury | Cancer<br>Heart<br>Pneumonia<br>Stroke<br>Injury |
| Male major causes of death | — | Heart<br>Injury<br>Cancer<br>Respiratory<br>Infectious | — | Heart<br>Cancer<br>Respiratory<br>Injury<br>Digestive | Cancer<br>Heart<br>Pneumonia<br>Stroke<br>Injury | Cancer<br>Heart<br>Pneumonia<br>Stroke<br>Injury |
| Female average hospital stay by cause (days): | — | — | — | | — | |
| Cancer | | | | 10.0 | | 6.9 |
| Heart | | | | 14.1 | | 9.2 |
| Respiratory | | | | 7.6 | | 4.8 |
| Digestive | | | | 5.8 | | 5.2 |
| Genitourinary | | | | 4.5 | | 4.4 |
| Musculoskeletal | | | | 10.6 | | 7.6 |
| Injury | | | | 10.1 | | 7.5 |
| All causes | | | | 9.9 | | 6.1 |
| Male average hospital stay by cause (days): | — | — | — | | — | |
| Cancer | | | | 11.5 | | 8.6 |
| Heart | | | | 10.6 | | 8.2 |
| Respiratory | | | | 6.8 | | 4.7 |
| Digestive | | | | 4.9 | | 4.5 |
| Genitourinary | | | | 5.2 | | 5.5 |
| Musculoskeletal | | | | 7.2 | | 6.4 |
| Injury | | | | 7.3 | | 5.2 |
| All causes | | | | 10.1 | | 7.8 |

**TABLE 6 (continued)**

| | Philippines | Thailand | Malaysia | Canada | Hong Kong | Singapore |
|---|---|---|---|---|---|---|
| Average hospital stay by cause - female to male ratio: | — | — | — | | — | |
| Cancer | | | | 0.87 | | 0.80 |
| Heart | | | | 1.33 | | 1.12 |
| Respiratory | | | | 1.12 | | 1.02 |
| Digestive | | | | 1.18 | | 1.16 |
| Genitourinary | | | | 0.87 | | 0.80 |
| Musculoskeletal | | | | 1.47 | | 1.19 |
| Injury | | | | 1.38 | | 1.44 |
| All causes | | | | 0.98 | | 0.78 |
| Female coronary bypass rate (per 10,000) | — | — | — | — | — | 18.1 |
| Male coronary bypass rate (per 10,000) | — | — | — | — | — | 49.0 |
| Coronary bypass rate - female to male ratio | — | — | — | — | — | 0.37 |
| Female % smokers | — | 1.9 | — | 23.3 | 2.9 | 3.2 |
| Male % smokers | — | 31.4 | — | 27.3 | 27.1 | 27.1 |
| % smokers - female to male ratio | — | 0.06 | — | 0.85 | 0.11 | 0.12 |
| Female % smokers by age, in years | — | 11-14 (0.0)<br>15-34 (0.8)<br>35-64 (0.4)<br>65+ (4.8) | — | 15-34 (29.3)<br>35-64 (23.1)<br>65+ (11.0) | 15-19 (1.3)<br>20-29 (4.3)<br>30-39 (2.0)<br>40-49 (2.7)<br>50-59 (1.5)<br>60+ (4.4) | 18-34 (4.4)<br>35-64 (2.1)<br>65-69 (7.0) |
| Male (%) smokers by age, in years | — | 11-14 (0.1)<br>15-34 (35.4)<br>35-64 (50.8)<br>65+ (45.0) | — | 15-34 (32.0)<br>35-64 (27.6)<br>65+ (12.9) | 15-19 (4.2)<br>20-29 (24.1)<br>30-39 (30.1)<br>40-49 (33.0)<br>50-59 (35.1)<br>60+ (24.2) | 18-34 (25.7)<br>35-64 (27.7)<br>65-69 (32.3) |

* "—" indicates no information was available.

## (1) Women's Health is an Important Issue in Public Health

Participants in all six countries mentioned the important role of women in the public healthcare system. Not only do women consult physicians and utilize healthcare services for their own medical and reproductive concerns, they typically manage and oversee healthcare for their families, including children, male partners, and elderly parents. This has been pointed out by previous researchers[3,25,26,27,28]. As one key informant in Hong Kong concisely summarized it, "women is centre of family nucleus, family health is nucleus of public health."

A second common theme was that women have traditionally been vulnerable in the society, their health needs have historically not been well met in the mainstream healthcare system, and as a result, women's health is affected by their inequitable position in the society.

In developing countries such as the Philippines, Thailand, and Malaysia, the main concern was the transition to new globalized economy resulting in more women working. Long hours of work put women's health at risk. Also, women bear children so they have health risks that men do not face (pregnancy-related hazards).

In developed countries such as Canada, Hong Kong, and Singapore, reproductive health concerns were considered things of the past, being replaced by emerging issues such as old-aged women and young unmarried women. The longer life expectancy makes women more likely to get chronic diseases and diseases of the old age than men. Also, women use more healthcare resources (doctor visits, hospital stays, drugs) than men[29]. As was pointed out in Canada, the “overwhelming demographic fact is that women live 6 years longer than men, so we must keep them healthy to save healthcare dollars.”

It is encouraging to see that women’s health is regarded as important by senior officials, researchers, and experts, with an emphasis on reproductive health (developing countries) or senior’s health (developed countries).

## (2) Special Initiatives (Programs) Relating to Women's Health

In the six countries, there were initiatives and programs in women’s health. Typically, the government provided reproductive health services (family planning, sex education, pregnancy health, STDs, HIV, abortion, infertility) and health screening (mammography and pap test) in major hospitals and health centers.

In developing countries, programs targeted more on using existing knowledge for prevention and control[30,31]. For example, in the Philippines, municipal officers disseminated pesticide information to women in villages. In Thailand, the government provided iron supplement tablets for female students and workers once a week for disease prevention. There were workplace and school programs for obesity management, including exercise and dancing.

In developed countries, programs focused more on generating knowledge[32,33]. In Canada, the government created five centers of excellence in 1996 to generate and synthesize new knowledge about women’s health. A newly created institute of gender and health funded women’s health research. In Hong Kong, universities conducted research in osteoporosis and elderly care especially among women. An equal opportunities commission (a nongovernment organization, NGO) was established to study issues on gender bias and employment discrimination. In Singapore, the government had a committee to study women’s health issues including osteoporosis, fracture, breast cancer, and cervical cancer.

Encouraging women’s health research is important because women’s health has been found to be different from men’s health in many body systems: cardiovascular[34,35], gastrointestinal[36], immune[37], resistance to HIV infection[38,39], and psychological[40]. There are sex differences in the metabolism of pharmaceutical agents[41]. Women who drink heavily are more likely than men to develop liver cirrhosis[6]. Recent research indicates that the health effect of being overweight is different in women and men[42]. Besides, more research should be conducted based on female subjects, as studies done on men do not necessarily apply to women. Equipment and medical devices traditionally designed based on the male body as the standard may not fit women[1]. Many psychotropic medications were tested on the male species even though these medications are clinically used mostly in women[41]. Knowledge generation is a necessary prerequisite for knowledge application.

## (3) Important Diseases (Health Conditions) Affecting Women's Health

As correctly pointed out by Canada, the list of important diseases among women depends on the indicator chosen, for example in terms of mortality (CVD, cancer, chronic obstructive pulmonary disease or COPD), in terms of potential years of life lost or PYLL (cancer, accident, CVD), or in terms of disability (arthritis, nervous system, limb problems). Nevertheless, cancer was identified by all participating countries as an important disease affecting women’s health. There was a difference between developing and developed countries in the other important diseases or health conditions (Table 2). In the checklist responses, the three developing countries (the Philippines, Thailand, and Malaysia) considered the following to be important: reproductive disorder, menopause-related condition, musculoskeletal disorder, car accident, and dental health. Developed countries (Canada, Hong Kong, Singapore) considered diabetes and osteoporosis to be important.

In developing countries, women’s diseases were mostly acute conditions such as pregnancy- and menopause-related health. For example, in Thailand, three most serious causes of maternal death at childbirth were mentioned: hemorrhage (14.0/100,000 live births), infection (8.4/100,000), and abortion (5.6/100,000).

In developed countries, chronic diseases predominated[43]. In Singapore, incidence of breast cancer was going up. Osteoporosis was becoming important because of the aging population. In Canada, certain chronic conditions were more prevalent among women than men, such as eating-related disorders, musculoskeletal problems, autoimmune disorders (arthritis and lupus), osteoporosis, chronic pain, domestic violence, stress, and mental health.

Natural reproductive processes such as menses, pregnancy, childbirth, and menopause cause physical and mental distress unique to women. Menopause increases women's risk of CVD and osteoporosis[1]. While women experience symptoms similar to men's, they are often faced with added difficult decisions related to pregnancy, such as the possibility of mother-to-fetus transmission[1] and fetal alcohol syndrome[6]. Furthermore, the longer female life expectancy suggests that healthy aging especially among women also needs to be better understood.

## (4) Important Risk Factors (Biological and Physical) Affecting Women's Health

All six countries considered lack of physical activity, high blood pressure, and diabetes to be important in women's health. For other types of risk factors, the Philippines, Thailand, and Malaysia considered environmental conditions, occupational exposures, and air and water quality to be important, whereas Canada, Hong Kong, and Singapore considered smoking and obesity to be important (Table 3).

In developing countries, with the changing economy, there was a rapid change in lifestyle to accept a higher proportion of women working outside their homes, resulting in a higher proportion of women smokers (job stress) and higher intake of processed food with higher preservatives and fat content (lack of time to prepare traditional home-made food). **“**Malaysians eat anything provided to them. They do not look at the ingredients. When the taste bud says it is good they buy.” As a result they ate too much processed food and food containing preservatives. The environment was deteriorating; poor housing and working environment, poor air and water quality.

In developed countries such as Canada, occupational exposure was not a problem as most women were in clerical jobs, not production jobs. “Poor working conditions are a men's problem.” Major concerns were smoking (among young women) and obesity. Due to advancing technology, women picked up a sedentary lifestyle, spending too much time in cars and in front of computers. Young girls dieted and did not eat enough, while working women had to eat in a hurry. In Hong Kong, “poor nutrition was not caused by lack of food but by overeating.” In Singapore, diabetes was a serious problem (prevalence in women 9.6%, men 8.5%).

Women are prone to certain health risks, e.g., mental disorders, accidents, STDs, suicidal thoughts, eating disorders, smoking, substance abuse, prescription drugs, and physical inactivity[1]. They have a lower self-image[9], higher levels of depression and mental health disorders[8,44], and a higher rate of psychiatric hospitalization[44]. While men commit suicide more frequently[44], women attempt suicide more often, but are more likely to fail in their attempts[10]. The media help create unrealistic social expectations about weight and appearance and subtly encourage women to attach undue importance to these. Among women there is widespread preoccupation with weight loss, for example, in Canada in 1995, 80–90% of women experience body image dissatisfaction[45], and among 12–14 year olds, 27% of girls and 14% of boys reported trying to lose weight[46]. There is a rising rate of anorexia and bulimia in women[45]. The complex interrelationship of multiple coexisting risk factors unique to women needs to be further understood.

## (5) Important Social Determinants (Social and Economic) Affecting Women's Health

Most countries cited problems of poverty (low income) and a caring role for family and friends (Table 4). In the Philippines, Thailand, and Malaysia, other problems mentioned were poor housing and vulnerability to unemployment. In Canada and Hong Kong, problems were single motherhood and violence against women.

In developing countries, the problems were mainly low income and poor access to healthcare. In Malaysia, for example, young women who had just married and pregnant women had difficulties in employment, as employers feared that the women would take maternity leave soon. In Thailand, women had problems trying to access health services, because they worked long hours, both at work and at home. Government clinics only opened from Monday–Friday, 8:30 a.m. to 4:30 pm. When women were free to go to the clinic, e.g., Saturday and Sunday, the clinics were closed. Private clinics were open all the time, but were expensive. On the other hand, single motherhood was not a problem because the divorce rate was low. Even for single mothers, there was an extended family so women could always go back to the family after divorce.

In developed countries, violence against women was considered serious and had a strong impact on mental health. Canadian statistics in 1993 indicated that half of Canadian women were victims of sexual or physical assault at least once beyond age 16[1]. Women’s access to healthcare was not a problem. “In Canada, men die more but women are more sick. Women go see doctors more and have better access to healthcare than men.” Legislation protecting women’s rights were in place (sexual harassment laws). In 2001, paid maternity leave increased from 6 months to 1 year. In Singapore, gender bias was not an issue, as there were many women top executives.

Women’s low income is a key problem. It is generally attributed to vertical gender segregation in the organizations (the existing male-dominated power structure preventing women to occupy power positions in organizations)[47], low education, and family responsibilities (reducing women’s number of years of work experience)[25].

The notion that in developed countries women have equal access to healthcare may not be entirely true. In Singapore, the coronary bypass rate was much lower in females than in males (Table 6). In another study, physicians responded differently to five medical complaints in men and women (chest pain, back pain, headache, dizziness, and fatigue), and ordered more work-up for male patients than female patients[48]. Among patients with cardiovascular radionuclide exercise scans, 40% of the men but only 4% of the women with abnormal test results were referred by their physicians for cardiac catheterization[49]. Even in a universal healthcare system (e.g., Canada), which provides universal access to physicians and hospital services, women are still less likely to have access to uninsured services such as drugs and dental care because they are less likely to be employed full time.

## (6) Important Challenges (Difficulties) Affecting Women's Health

Most of the countries agreed that the most important challenge in women’s health was informing, educating, and empowering people about women’s health issues (Table 5). The question was not only educating women, but men as well — “In women’s health seminars, we often see women but no men attending.” In the Philippines and Thailand, other challenges were conducting research for new insights and innovative solutions, and lack of adequate funding for projects and programs. In Canada and Hong Kong, challenges included monitoring women’s health status, investigating women’s health problems and hazards, and mobilizing partners to identify and resolve women’s health problems.

In developing countries, the major challenge was lack of money and inadequate political involvement of women. As there were very few female politicians, women’s health issues were overlooked. More NGOs were needed to promote women’s rights.

In developed countries, funding was not considered a major problem. In Canada, there was “lack of horizontality, and a long-term, holistic strategy to women’s health.” There was insufficient monitoring and investigation of women’s health problems (e.g., arthritis, musculoskeletal disorders, mental health). “These diseases do not kill people, so do not get much attention.” There were not enough researchers with an interest and knowledge in women’s health. “Our healthcare system does not understand that babies, girls, women, seniors have different health needs.” On the other hand, there was an encouraging trend of change of women from patient to consumers. “They are now doctor shopping. They look for health information on the Internet." In Singapore, mammography and pap tests were provided to women for free. The challenge was in educating women so they participate in regular screening.

Policy makers and care providers need to understand the challenges and study the differences in women's and men's health profiles. In Canada, women outlive men by 6 years (life expectancy at birth for women 81 years, for men 75 years, in 1994)[44]. Women's death rate is lower than men in all age groups[50]. Between the ages of 20 and 44, men's likelihood of dying is more than twice that of women, mostly attributable to external causes such as motor vehicle accidents and suicide[51]. Despite a lower death rate, women suffer more than men from chronic conditions, particularly migraines, allergies, arthritis, rheumatism, and back and limb problems[44]. In infancy and childhood, girls use fewer health services than boys. But once beyond childhood, Canadian women make greater use of a wide range of health services[1]. Due to women's long life expectancy, their later life is often characterized by isolation, disability, and health problems[50].

## RECOMMENDATIONS

While it may not be possible to ensure equality in health outcomes (e.g., life expectancy or morbidity rates) between women and men due to their biological (sex) differences, it is possible to ensure that the two groups have equal access to the resources which they need to realize their potential for health; in other words, to eliminate their social (gender) differences[3]. From results of this study, gender equity in health is achievable through a number of ways:

### (1) Surveillance

1. Mandate sex-disaggregated data and gender-based analysis. Sex-disaggregated data (data cross-classified by sex) are essential inputs into gender-based analysis[26]. In developed countries, health, risk, and intervention data are often collected for both women and men, but are often not analyzed by gender. In developing countries, sex-disaggregated data may even not be available.
2. Develop objective and subjective indicators for gender statistics. Gender statistics, e.g., wage gaps, certain causes of mortality and disability, access to health education and care, must be used to identify the differing roles and needs of women and men, and the differing impacts of programs and policies on women and men, to improve allocation of resources[12,13,26].
3. Conduct women's opinion surveys on women's issues and health needs. Regular surveys of women on their issues and concerns, such as unpaid work, violence, and reproductive healthcare, will serve to help understand the problems and feasibility of practices among women. For example, a female key informant in this study mentioned the "mindless promotion of breast feeding in public places and the workplace, which is not feasible and not acceptable by many women."
4. Develop new essential surveillance programs especially related to emerging women's health issues, such as HIV/AIDS and violence against women, etc.[52].

### (2) Research

5. Conduct health research (etiology, diagnosis, and treatment) for women and men equally, recognizing that they are biologically and socially different. Traditional extrapolation of research results from men to women should be stopped.
6. Promote research to identify and resolve particular issues affecting women's health, e.g., mental health, fatigue, family violence, osteoporosis, abortion, eating disorders, smoking, and drug overdose.
7. Promote research on the role ascribed by biology and society to women (e.g., child bearer, caregiver) and its associated consequences (e.g., double workload, social inequality). It is important to understand why women have a longer average life expectancy than men and why they do not necessarily lead healthier lives[5].

## (3) Programs and Policies

8. Maintain a network of stakeholders (governments, researchers, advocacy groups, individuals) to oversee the continuing improvement of women's health. Proper infrastructures may have to be established.
9. Increase women's role in decision making. Women's health issues will get more attention if there are more "woman politicians, especially a woman minister of health or head of state." In many countries, women who have taken the challenge of political life often find themselves vulnerable and subjected to various unwanted challenges, such as the media's intense scrutiny and inaccurate portrayal. Political parties and organizations do not easily accept or promote women in their caucus[25]. Ways must be explored to increase women's participation in politics and decision making.
10. Resolve women's dual role at home and at work. Women are traditionally perceived to have a primary role at home as wives and mothers, yet, out of sheer economic necessity, women also go out to join the employment market. Extended hours of work lead to stress and have a negative impact on women's health. A change is needed in the societal perception in the traditional division of labor between men and women, both inside and outside the home[7]. In addition, ways to slow down the accelerating pace of the modern society (e.g., 4-day work week) need to be explored to give people, especially women, time to look after their health.
11. Reduce women's poverty and increase their economic empowerment. Poverty can affect both women and men, but women often suffer additional disadvantage due to discrimination[5]. In Hong Kong in 1996, an average working woman earned about 76% of a working man[25]. Women's health is improved if they do not need to become increasingly worried with earning a daily living and making ends meet. Increased income also means increased ability to purchase health care.
12. Mobilize men to partner in resolving women's health issues. In informing, educating, and empowering people about women's health issues, the key is to mobilize men. Women's health seminars should see both women and men attending. "Women's health issue is not only a women's issue; it is also a men's issue." For example, men's participation is needed to reduce and finally eliminate violence against women.
13. Guarantee women, by written policy, equal access to all aspects of healthcare as men, in view of their biological, social, and economic barriers.
14. Increase effort to educate teenage girls not to start smoking. In developed countries such as Canada, unprecedented numbers of women are dying from lung cancer, due to the alarming rate at which adolescent girls are taking up smoking[53]. In 1994, for the first time, lung cancer surpassed breast cancer as the leading cancer killer of Canadian women[54]. This trend may hit developing countries in a few years.
15. Encourage women of all ages to perform adequate physical activity and to avoid overweight.
16. Encourage healthy eating for women of all ages.
17. Encourage women in certain age groups to participate in routine medical examination and screening for certain types of chronic diseases (e.g., cervical cancer and breast cancer).

## PREDICTED TRENDS OF WOMEN'S HEALTH IN DEVELOPING COUNTRIES

Because of globalization of the economy, it is expected that women's health in developing countries will follow a similar pattern in women's health in developed countries. From this study, a number of future scenarios can be suggested to the developing countries to prepare for predicted forthcoming changes in women's health patterns based on the experience of the developed countries. The pattern of women's health will shift from reproductive health (currently in developing countries) to senior's health (similar to developed countries); from acute conditions such as pregnancy- and menopause-related health to chronic conditions such as cancer, diabetes, and osteoporosis; from knowledge application such as rural health and occupational health practices to knowledge generation such as women's health research; from musculoskeletal problems and occupational and environmental contaminants to lack of physical activities associated with modern technology such as car and computer; from low income and poor access to healthcare to violence against women and single motherhood; and from lack of funding for programs in women's health to lack of research into the needs of women at various stages of life.

## ACKNOWLEDGMENTS

Funding support for this study was provided by the Southeast Asia Gender Equity Program (SEAGEP) of the Canadian International Development Agency (CIDA).
